# Cryoballoon Pulmonary Vein Isolation and Roof and Posterior Wall Debulking Using Navik 3D™: A New Technique for Atrial Fibrillation Ablation

**DOI:** 10.19102/icrm.2020.110105

**Published:** 2020-01-15

**Authors:** Imran Niazi, Lynn Erickson, Amir Chaudhari, Mohamed Djelmami-Hani

**Affiliations:** ^1^Aurora Cardiovascular Services, Aurora Sinai/Aurora St. Luke’s Medical Centers, University of Wisconsin School of Medicine and Public Health, Milwaukee, WI, USA

**Keywords:** Ablation, atrial fibrillation, cardiac mapping, cryoballoon, Navik 3D

## Abstract

The success rates of traditional endocardial ablation techniques for managing atrial fibrillation remain modest. Recently, the performance of posterior wall ablation in conjunction with pulmonary vein (PV) isolation (PVI) has been reported to increase the chance of success following endocardial ablation. We report a systematic approach for the isolation of the PVs and ablation of the left atrial roof and posterior wall using a cryoballoon guided by the novel Navik 3D™ mapping system (APN Health LLC, Waukesha, WI, USA) and offer preliminary data including procedure, fluoroscopy, and cryoablation times for review. Patients (n = 52) aged 63 years ± 10 years with paroxysmal (n = 42), persistent (n = 11), or chronic (n = 2) atrial fibrillation underwent cryoballoon ablation for PVI and/or the left atrial roof, posterior wall, anterior ganglion plexi (GP), or mitral isthmus line. Lesions were accurately delivered to the PVs, left atrial roof, posterior wall, anterior GP, or mitral isthmus line as appropriate. Acute PVI was achieved in 98% of all patients, and eight (15%) required direct current cardioversion to restore sinus rhythm at the end of the procedure. The mean ± standard deviation procedure, fluoroscopy, and cryoballoon ablation times were 149 minutes ± 39 minutes, 33 minutes ± 30 minutes, and 41 minutes ± 14 minutes, respectively. The Navik 3D™ mapping system is believed to be the only available mapping system that allows for the visualization and location of the cryoballoon in three dimensions, enabling the operator to deliver contiguous, overlapping lesions on the roof and posterior wall of the left atrium. It also facilitates precise measurement of the distance between the esophageal temperature probe and the cryoballoon, thereby helping to avoid freezing damage to the esophagus.

## Introduction

Pulmonary vein (PV) isolation (PVI) remains the cornerstone of atrial fibrillation (AF) ablation, but the success rate remains modest, particularly in patients with a dilated left atrium and persistent AF.^1–3^ Since the posterior left atrial wall is embryologically akin to the PVs,^4^ debulking of the posterior wall and left atrial roof has been proposed as an adjunct technique in difficult cases.^5^ Hybrid ablation, in which endocardial PVI is supplemented with minimally invasive surgical epicardial posterior wall ablation, has been shown to improve the success rates in refractory paroxysmal, persistent, and long-standing persistent AF.^6,7^ Interestingly, supplementing PVI with radiofrequency (RF) rooflines and mitral isthmus lines does not seem to reduce the recurrence rate, as noted in the Substrate and Trigger Ablation for Reduction of AF Trial Part II (STAR AF II).^8^

Recently, Aryana et al. achieved improved success rates with cryoballoon ablation of the posterior wall/roof debulking in addition to PVI in patients with persistent AF.^9^ This outcome might be due to the fact that cryoballoon lesions have a much larger footprint than the point-by-point RF lesions used to create rooflines and mitral lines. Others have reported increased success with a supplemental RF posterior wall “box” lesion set,^3,5^ albeit with an increased risk of freezing injury to the esophagus.^10^ This, in our experience, does not seem to occur with cryoballoon ablation of the posterior wall using the Navik 3D™ three-dimensional (3D) mapping system (APN Health LLC, Waukesha, WI, USA). The exact distance between the esophageal temperature probe and the distal marker on the cryoballoon can be calculated, and thermal effects on the esophagus can be mitigated by subtle repositioning such that the distal cooling surface (ie, the front half) of the balloon is oriented away from the esophagus and does not press the atrial wall into the esophagus.

We have developed effective techniques for cryoballoon-based posterior wall, roof, mitral isthmus, and anterior wall ablation employing the Navik 3D™ mapping system. With this system, two-dimensional fluoroscopic images are accurately converted into 3D maps. The cryoballoon can be visualized and virtually located, ensuring rapid and complete obliteration of electrograms on the roof and posterior wall with overlapping lesions, preventing gaps in ablation lines or areas. The Navik 3D™ mapping system is the only open-platform mapping system currently available and can be used to locate all catheters and esophageal probes in three dimensions without limitations on manufacturer, which significantly reduces the cost of the procedure. In the current report, we illustrate these techniques and describe the utility of this system.

The Navik 3D™ device can be used to report the 3D location (X, Y, and Z coordinates) of the distal marker on the cryoballoon and the esophageal temperature probe. Given these coordinates, the device can then compute the distance from the distal marker on the cryoballoon to the esophageal temperature probe. Although Navik 3D™ is a fluoroscopy-based system, preliminary tests of the system note that its use did not significantly increase fluoroscopic exposure when compared with recently published data on the subject of PVI plus posterior wall ablation using the cryoballoon.^11^

Navik 3D™ also allows for the construction of a 3D geometric representation of the left atrium using the cryoballoon, just as mapping catheters can be used to create the same using other mapping systems. Its open-platform design, which removes the need to involve these other systems, allows for significant cost reductions. No proprietary surface electrodes need to be applied nor are there proprietary ablation catheters to connect to enable the system. Since there is no recording electrode on the cryoballoon, there are no other mapping systems currently available that allow the cryoballoon to be located in 3D or to be used for mapping purposes.

We herein describe our experience tracking the cryoballoon and esophageal temperature probe to assist in the ablation of the PVs and/or the left atrial roof, posterior wall, anterior ganglion plexi (GP), and mitral isthmus line.

## Methods

### Study population

We included 52 patients aged 63 years ± 10 years with paroxysmal (n = 42), persistent (n = 11), or chronic (n = 2) AF who underwent cryoballoon ablation using only the Navik 3D™ system between March 2018 and April 2019 for PVI and/or cryoablation of the posterior wall, left atrial roof, anterior wall GP, and mitral isthmus line in this research. Baseline characteristics are outlined in **Table 1**.

### System training and general use

Typically, a hospital-employed electrophysiology (EP) laboratory technician operates the Navik 3D™ system under the guidance of the electrophysiologist performing the ablation procedure. Both the EP laboratory technician and the EP physician can view the Navik 3D™ display throughout the procedure. Initial system training for those operating the system consisted of a roughly 60-minute-long slide presentation of the system, discussion of the Navik 3D™ theory of operation, instruction on workflow and key functions, and review of the user manual, with any questions answered by a representative of the manufacturer. Next, the operator performed five procedures under the mentorship of an experienced operator prior to being approved to use the system independently.

### Pulmonary vein isolation with Navik 3D™ guidance

**Figure 1:**
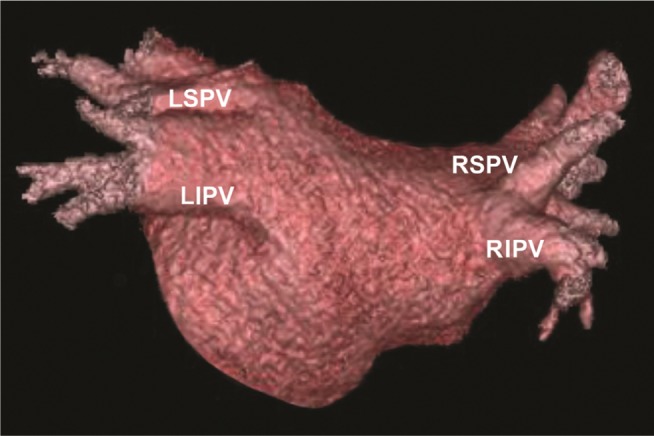
Computed tomographic reconstruction of the left atrium, posterior view.

**Figure 2:**
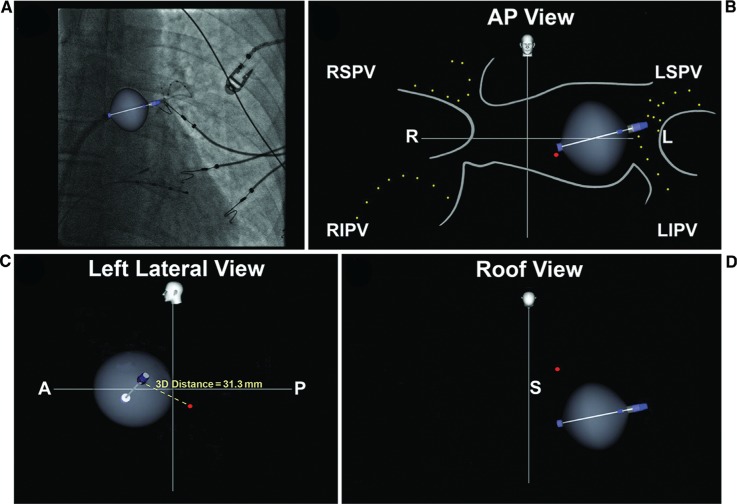
Cryoballoon in the antrum of the LSPV as seen on a Navik 3D™ image. The long axis (pin) and direction (blue tip) of the cryoballoon and the Achieve electrodes (yellow dots) are shown. The red dot indicates the esophageal electrode. **A:** Navik 3D™ fluoroscopy image in AP view. **B:** Navik 3D™ AP view showing stylized left atrial contours, PVs, and corresponding Achieve catheter (Medtronic, Minneapolis, MN, USA). The cryoballoon has been inflated in the antrum of the LSPV. **C:** The distance between the cryoballoon tip and the esophageal temperature probe is computed using Navik 3D™. **D:** Cranial (roof) view showing the cryoballoon positioned in the LSPV antrum and esophageal electrode location. A: anterior; L: left; P: posterior; R: right; S: superior; AP: anteroposterior; LIPV: left inferior pulmonary vein; LSPV: left superior pulmonary vein; RIPV: right inferior pulmonary vein; RSPV: right superior pulmonary vein.

**Figure 3:**
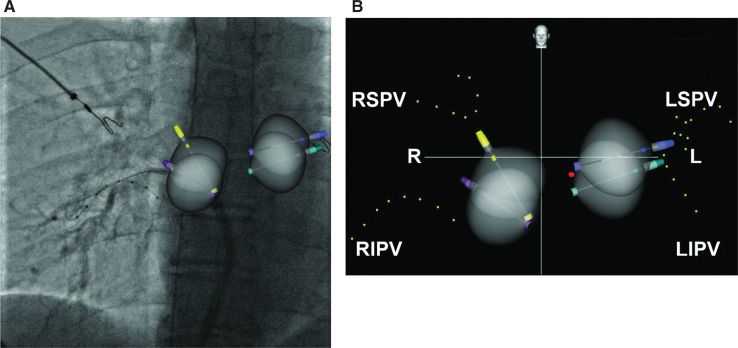
AP view of the Navik 3D™ screen following cryoballoon isolation of all four PVs. The location and orientation (indicated by the pins) of the cryoballoon in the antrum of each vein are marked. Achieve electrodes (yellow dots) mark the positioning of the PVs. The red dot represents the esophageal temperature probe location. L: left; R: right; LIPV: left inferior pulmonary vein; LSPV: left superior pulmonary vein; RIPV: right inferior pulmonary vein; RSPV: right superior pulmonary vein.

The PVs were cannulated with the Achieve multielectrode circular catheter (Medtronic, Minneapolis, MN, USA) and PV occlusion was confirmed with contrast injection after advancing the cryoballoon into the target vein. A preoperative computed tomography scan of the PVs may be helpful to assess the vein location but is not necessary **(Figure 1)**. The Achieve catheter (Medtronic, Minneapolis, MN, USA) was utilized by the Navik 3D™ system to mark the location of each PV, and the esophageal electrode was also located in 3D, utilizing the radiopaque temperature sensor marker. In this way, the distance and orientation of the cryoballoon in relation to the esophageal temperature probe were computed and visualized on the map **(Figure 2)**. The location and orientation of the cryoballoon were also marked by Navik 3D™ in each vein **(Figure 3)**. Two fluoroscopic views are required for each location on the map, separated by about 20 degrees. We utilized the left anterior oblique 20-degree and anteroposterior (AP) views for the left PVs and the right anterior oblique 20-degree plus AP views for the right PVs for the sake of convenience.

PVI was performed with three-minute initial and two-minute confirmatory lesion creation in each vein; isolation was documented with the Achieve multielectrode catheter (Medtronic, Minneapolis, MN, USA) and confirmed with entrance/exit block.

### Left atrial roof and posterior wall ablation

**Figure 4:**
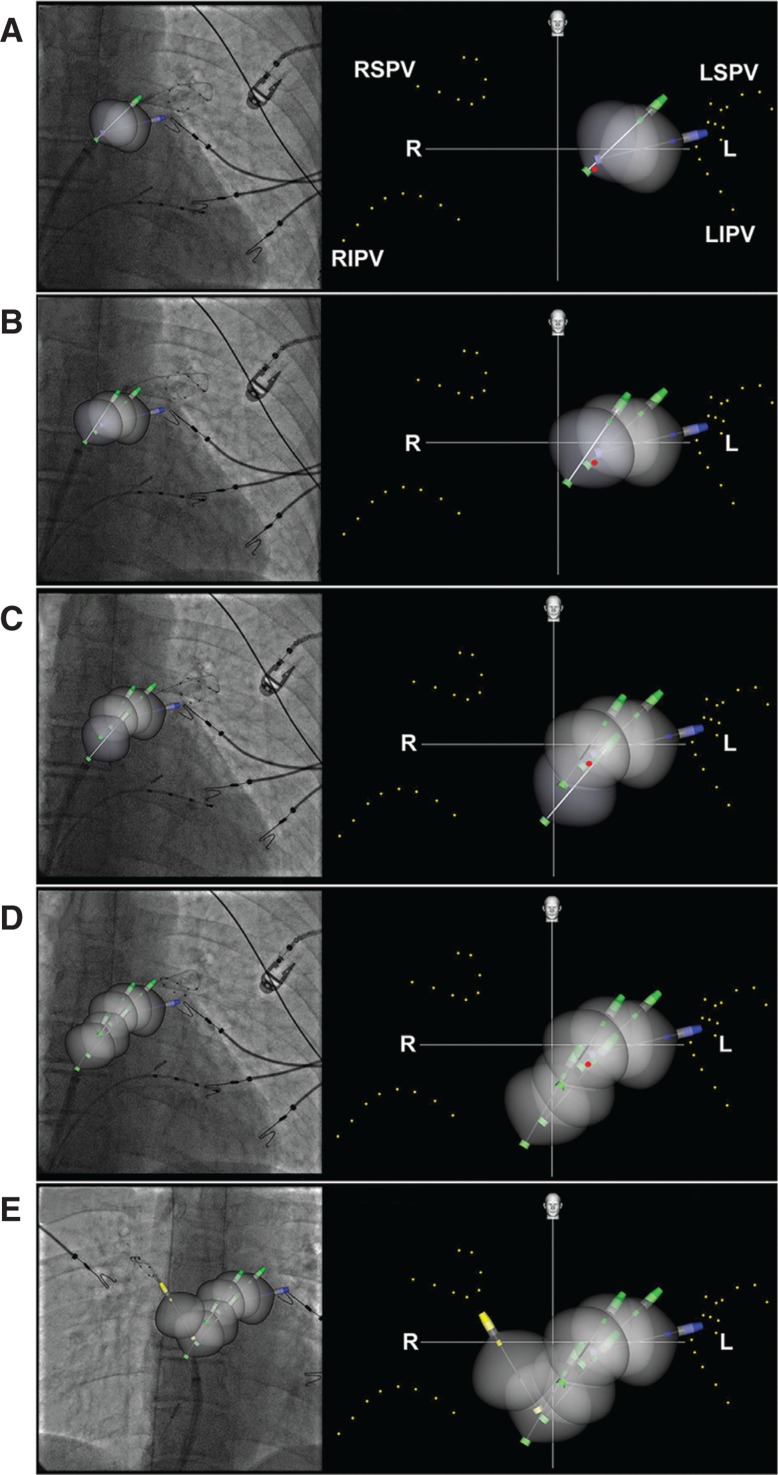
Creation of the roofline in the AP view with the Navik 3D™ fluoroscopy image on the left and corresponding Navik 3D™ map on the right. Achieve electrodes (yellow dots) mark the location of the PVs and the esophageal temperature probe (red dot). **A:** The cryoballoon was withdrawn by half a balloon length from the LSPV antrum and placed against the roof, at which point, the pin color changes to green. **B:** Roof lesion no. 2. The balloon was withdrawn and deflected upwards and the FlexCath sheath (Medtronic, Minneapolis, MN, USA) was relaxed with clockwise rotation. **C:** Roof lesion no. 3. The balloon was withdrawn further. The cryoballoon location was lower, owing to the left-to-right slope of the left atrial roof. **D:** Fourth and final left roof lesion. The cryoballoon was withdrawn further with clockwise rotation, while the Achieve catheter (Medtronic, Minneapolis, MN, USA) remained in the LSPV to act as a steadying rail. **E:** Cryoballoon lesion in the antrum of the RSPV (yellow pin). The entire roof was covered by contiguous lesions, and a final lesion proximal to the RSPV was not required in this patient. L: left; R: right; LIPV: left inferior pulmonary vein; LSPV: left superior pulmonary vein; RIPV: right inferior pulmonary vein; RSPV: right superior pulmonary vein.

We used the cryoballoon to rapidly and completely obliterate all electrical activity over the left atrial roof and posterior wall. After left superior PV (LSPV) antral isolation, the Achieve catheter (Medtronic, Minneapolis, MN, USA) was retained in the LSPV and the cryoballoon was withdrawn slightly (about half a balloon length). The curve of the FlexCath Advance steerable sheath (Medtronic, Minneapolis, MN, USA) was released, and the cryoballoon was directed against the contiguous portion of the roof **(Figure 4A)**. The cryoballoon itself may be deflected superiorly using its intrinsic controls if necessary. The Achieve catheter (Medtronic, Minneapolis, MN, USA) acted as a rail and steadied the cryoballoon for this and subsequent lesions. A three-minute cryoablation lesion was delivered. A temperature nadir of less than −35°C was considered as an indication of good contact. In our experience, three-minute lesions with good contact and good temperature drop are essential for the durable obliteration of all electrical activity in the contacted area.

**Figure 5:**
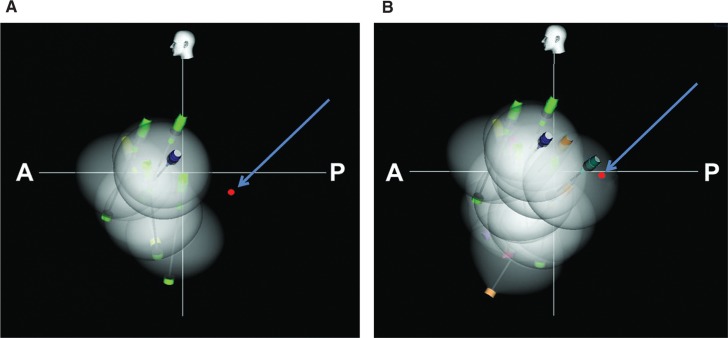
Navik 3D™ maps. **A:** Left lateral view following left superior PV and roof lesions. Note the esophageal electrode location (red) and its distance from the lesions. **B:** Left lateral view after complete lesion set. The left inferior PV lesion overlapped the esophageal electrode and was associated with an esophageal temperature drop to 31°C. A: anterior; P: posterior.

Subsequently, with the cryoballoon inflated, the FlexCath sheath (Medtronic, Minneapolis, MN, USA) was rotated clockwise, the balloon was withdrawn by half a balloon length, and a second roof lesion was delivered **(Figure 4B)**. Third; fourth; or, more rarely, more roof lesions may be required to cover the entire roof up to the right superior PV (RSPV) **(Figures 4C and 4D)**. Next, the RSPV antrum was ablated in the usual way **(Figure 4E)**. Following RSPV antral ablation, the cryoballoon was inflated and pulled back from the RSPV slightly and the FlexCath sheath (Medtronic, Minneapolis, MN, USA) was rotated counterclockwise while retaining the Achieve catheter (Medtronic, Minneapolis, MN, USA) within the RSPV. A lesion delivered in this location completed the right side of the roofline. On occasion, this lesion may not be necessary if sufficient overlap is achieved with just the roof and RSPV lesions. It is noteworthy that certain lesions may overlap the esophageal electrode, which may be associated with a drop in esophageal temperature **(Figure 5)**.

**Figure 6:**
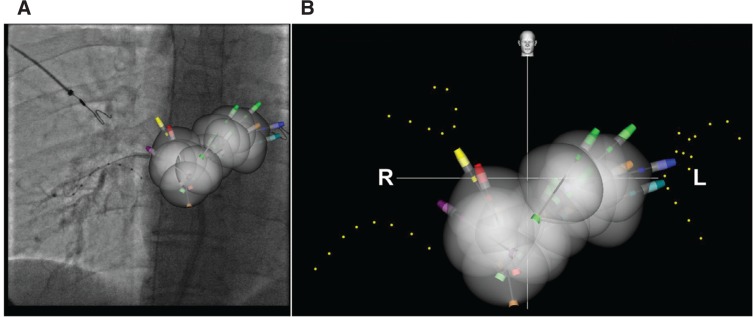
**A:** Navik 3D™ fluoroscopic and **B:** Navik 3D™ map reconstruction in the AP view showing cryoballoon ablation of all four PVs left atrial roof, and posterior wall.

Posterior wall ablation was started with antral cryoablation of the left inferior PV (LIPV). The Achieve catheter (Medtronic, Minneapolis, MN, USA) was retained in the LIPV and the cryoballoon was inflated and withdrawn by half a balloon length (about 10–15 mm), then directed against the contiguous posterior wall by clockwise rotation of the FlexCath sheath (Medtronic, Minneapolis, MN, USA). Increasing the curvature of the FlexCath sheath (Medtronic, Minneapolis, MN, USA) allows the operator to direct the balloon against the posterior wall rather than the roof. As elsewhere, three-minute lesions were delivered on the posterior wall. For subsequent lesions, the process was repeated with the Achieve catheter (Medtronic, Minneapolis, MN, USA) retained in the LIPV to act as a stabilizing rail. Several lesions—usually three to four—may be required to cover the entire posterior wall, depending on the size of the atrium. Following right inferior PV (RIPV) isolation, a right posterior wall lesion was delivered by withdrawing the cryoballoon while rotating it counterclockwise with the Achieve catheter (Medtronic, Minneapolis, MN, USA) retained in the RIPV for stability. This completed the debulking of the entire posterior wall **(Figure 6)**. Roof and posterior wall ablation can be checked by the Achieve circular catheter and CardioLab (GE Healthcare, Chicago, IL, USA) or a similar EP recording system.

### Ablation anterior to the RSPV

The anterior wall contiguous to the RSPV is richly innervated with GPs and is frequently the site of complex and fractionated electrograms both during AF and in normal sinus rhythm. The ablation of this area may be accomplished by withdrawing the cryoballoon and exerting clockwise torque on the FlexCath sheath (Medtronic, Minneapolis, MN, USA) to bring the cryoballoon into intimate contact with the contiguous anterior wall. Creation of a single three-minute lesion is usually sufficient to debulk this area.

## Results

### Procedure results

Cryoablation lesions were performed in all or some of the following: PVI, left atrial roof, posterior wall, anterior GP, mitral isthmus line, and/or complex electrograms **(Table 2)**. Isolation of the PVs was achieved in 51 (98%) of the patients, as confirmed with entrance/exit block using bidirectional pacing. The endpoint for effective debulking of the posterior wall and roof was the achievement of no residual electrical activity of more than 0.2 Mv in amplitude. Since pacing is not possible when electrograms have been completely obliterated, we could not check for exit block. The total procedure time was 149 minutes ± 39 minutes, while fluoroscopy time was 33 minutes ± 30 minutes and cryoballoon ablation time was 41 minutes ± 14 minutes. Direct-current cardioversion was required in eight (15%) of patients to restore sinus rhythm at the end of the procedure.

**Figure 7:**
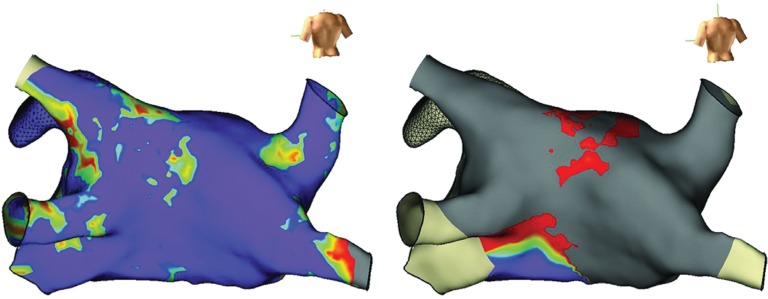
Voltage map of the left atrium in the posterior view in a representative patient obtained with the EnSite Velocity system and Advisor HD Grid catheter (Abbott Laboratories, Chicago, IL, USA); voltage settings were 0.2 to 1.5 Mv. **A:** Prior to ablation. **B:** After full lesion set.

Early on in our experience, we used a separate 3D mapping system to confirm results in each patient. **Figure 7** represents a voltage map acquired with the EnSite Velocity (Abbott Laboratories, Chicago, IL, USA) system prior to and after the complete cryoablation lesion set was established in a representative patient. Mapping was performed with the Advisor HD Grid (Abbott Laboratories, Chicago, IL, USA) multielectrode catheter and a total of 13,000 points were acquired. Voltage settings were 0.2 to 1.5 Mv. The posterior wall was effectively debulked and a complete roofline was established.

## Discussion

The technique we describe is highly useful in delivering effective and contiguous lesions on the roof and posterior wall. After cryoablation, the absence of electrograms on the roof and posterior wall can be confirmed by mapping with the Achieve catheter (Medtronic, Minneapolis, MN, USA). The ability to locate cryoballoon ablation lesions in real time during the procedure aids in the accurate delivery of such lesions and may reduce the number of lesions that would be given otherwise without using Navik 3D™.

Although Aryana et al. discussed using two-minute freezes for PVI and posterior wall ablation,^9^ our center traditionally uses an initial three-minute freeze with another bonus freeze performed to ensure isolation. Continuous monitoring of esophageal temperature during the procedure, along with the capability to map the esophagus and cryoballoon lesion location using Navik 3D™ during the procedure, provides useful information to help avoid injury to the esophagus during cryoablation.

Although the system is fluoroscopy-based, we reported fluoroscopy and procedure times that are similar to those of other institutions obtained using different cardiac mapping techniques. In this population, fluoroscopy and procedure times were 33 minutes and 149 minutes, respectively. A recent multicenter study of patients undergoing cryoablation for PVI and the posterior wall reported fluoroscopy and procedure times of 28 minutes and 188 minutes, respectively.^9^ It is important to note that 46 (88%) of our patients received cryoablation that may have included the roofline, posterior wall, anterior GP, and/or the mitral isthmus line in addition to all having received PVI. Of note, fluoroscopy is the only method currently available to locate or visualize the cryoballoon within the heart.

## Conclusion

The Navik 3D™ system is a new, open-source 3D mapping system that is uniquely suited for anatomic cryoablation strategies for AF and left atrial flutter. This characteristic contributes to this technology’s simplicity and low cost. Navik 3D™ permits exact localization of the cryoballoon in relation to the esophagus and may help to prevent injury to that structure. Debulking of the posterior wall and roof in persistent AF is performed easily, quickly, and safely, and the open-source architecture allows for significant cost savings.

## Figures and Tables

**Table 1:** Baseline Characteristics

Characteristic	n = 52
Age	63 ± 10 years
Male gender	37 (67)
Body mass index	30 ± 6 kg/m^2^
CHA_2_DS_2_-VASc score	2 ± 2
Diabetes mellitus	6 (12)
Hypertension	35 (67)
Coronary artery disease	19 (37)
Chronic obstructive pulmonary disease	6 (12)
Sleep apnea	12 (23)
CPAP use	6 (50)
Left ventricular ejection fraction	58% ± 19%
Left atrial volume	36 ± 19 mL/m^2^
Number of failed antiarrhythmics	0.9 ± 0.9
Patients with one or more prior cardioversions	13 (25)
Number of prior cardioversions	0.5 ± 1

CPAP: continuous positive airway pressure.

Values are presented as means ± standard deviations or as n (%).

**Table 2:** Lesions Created Using the Cryoballoon

Cryoablation Lesions Applied	Number of Patients Who Received the Lesion Set
PVI, roofline, posterior wall, anterior left atrium GP	17
PVI, roofline, and posterior wall	12
PVI and roofline	12
PVI alone	6
PVI, roofline, posterior wall, mitral isthmus	2
PVI, roofline, and complex electrograms	1
PVI and posterior wall	1
PVI and anterior left atrium GP	1

GP, ganglion plexi; PVI: pulmonary vein isolation.
